# Robot-assisted partial nephrectomy in contemporary practice

**DOI:** 10.3389/fonc.2012.00213

**Published:** 2013-01-11

**Authors:** Youssef S. Tanagho, Sam B. Bhayani, Robert S. Figenshau

**Affiliations:** Division of Urologic Surgery, Washington University School of MedicineSt. Louis, MO, USA

**Keywords:** robotic partial nephrectomy, robot-assisted partial nephrectomy, robotic surgery, minimally invasive surgical procedures, renal cell carcinoma, partial nephrectomy, outcomes assessment

## Abstract

Laparoscopic renal surgery is associated with reduced blood loss, shorter hospital stay, enhanced cosmesis, and more rapid convalescence relative to open renal surgery. Laparoscopic partial nephrectomy (LPN) is a minimally invasive, nephron-sparing alternative to laparoscopic radical nephrectomy (RN) for the management of small renal masses. While offering similar oncological outcomes to laparoscopic RN, the technical challenges and prolonged learning curve associated with LPN limit its wider dissemination. Robot-assisted partial nephrectomy (RAPN), although still an evolving procedure with no long-term data, has emerged as a viable alternative to LPN, with favorable preliminary outcomes. This article provides an overview of the role of RAPN in the management of renal cell carcinoma. The clinical indications and principles of surgical technique for this procedure are discussed. The oncological, renal functional, and perioperative outcomes of RAPN are also evaluated, as are complication rates.

## INTRODUCTION

While the gold standard treatment of renal tumors was previously radical nephrectomy (RN), investigation over the past decade has demonstrated the surgical feasibility and equivalent oncologic efficacy of partial nephrectomy (PN) for the management of small renal masses (SRM; Fergany et al., 2000). With mounting evidence indicating that overtreatment of renal masses with RN is associated with increased risk of chronic renal insufficiency, cardiovascular events, and premature deaths (Go et al., 2004; Thompson et al., 2008; Weight et al., 2010), the American Urological Association guidelines now explicitly endorse PN as the standard of care for managing T1a renal tumors and as an alternative treatment option for T1b tumors (American Urological Association Education and Research, 2009). Reflecting this paradigm shift, PN utilization has increased considerably at many centers of excellence over the past decade, approaching 90% for T1a tumors at some centers (Thompson et al., 2009).

With the rapid dissemination of minimally invasive technology within the urologic community, laparoscopic PN (LPN) and, more recently, robot-assisted PN (RAPN) have emerged as viable alternatives to open PN (OPN) for the management of suspected renal malignancy. The long-term oncological and functional outcomes of LPN are similar to those of OPN (Gill et al., 2007; Lane and Gill, 2010), with the potential benefits of reduced blood loss (estimated blood loss, EBL), shorter hospital stay (length of hospital stay, LOS), superior cosmesis, and more rapid convalescence. However, LPN remains technically demanding, necessitating substantial technical expertise to achieve adequate tumor resection and renorrhaphy while minimizing ischemia times. Despite the development of novel techniques to facilitate LPN, the protracted learning curve associated with this procedure has hindered its dissemination into general practices in the U.S. and may, indeed, contribute to the underutilization of PN (Hollenback et al., 2006).

Among its potential advantages, robotic technology offers high-definition three-dimensional visualization, a broad range of wristed-instrument motion, and scaling of surgeon movements. RAPN appears to have a shorter learning curve than LPN (Mottrie et al., 2010) and, accordingly, may facilitate and promote the utilization of minimally invasive nephron-sparing surgery (NSS). We review the technique and outcomes of RAPN, assessing its current role and future prospects for the management of renal masses.

## SET UP

For the inexperienced robotic renal surgeon, judicious patient selection is critical. The lack of haptic feedback and reliance on the bedside assistant can present challenges unique to RAPN. Patients ideally suited for initial RAPN procedures include those with non-hilar, exophytic T1a lesions, uncomplicated vascular anatomy, and a normal contralateral kidney.

### EQUIPMENT

Robot-assisted partial nephrectomy is performed using the da Vinci surgical system (Intuitive Surgical Inc., Sunnyvale, CA, USA). Three robotic instruments are frequently used during RAPN – the Monopolar Curved Scissors in the dominant hand and ProGrasp forceps in the non-dominant hand; these are exchanged for robotic needle drivers during renorrhaphy. The ProGrasp’s blunt tips are suited for dissection of vessels and tumor and can be used to apply robotic bulldog clamps (Scanlan International, St. Paul, MN, USA) and manipulate the robotic ultrasound probe, if needed. The PK (PlasmaKinetic) dissecting forceps, Maryland bipolar forceps, and robotic hook are other potentially useful instruments, which can aid in precise dissection and cauterization of small vessels. The scrubbed assistant employs conventional laparoscopic instruments to provide suction and countertraction via the assistant port. The fourth arm can be used with a retracting device to improve exposure, although instrument collisions may increase with its use, especially in patients with small torsos.

### PATIENT POSITIONING AND PORT PLACEMENT

Patients undergoing transperitoneal RAPN are placed in a 75° modified flank/lateral decubitus position with the pathologic side up. A 90° full flank/lateral decubitus position is used for retroperitoneal RAPN (Gettman et al., 2004; Dulabon and Stifelman, 2011; White et al., 2011).

The transperitoneal approach is the most commonly used approach for RAPN. The most widely utilized transperitoneal camera/trocar configuration places the camera medial and superior to the umbilicus; a 30° downward-angled lens is used. Two 8-mm trocars for the robotic arms are placed just cephalad of the anterior superior iliac spine and inferior to the costal margin in the mid-axillary line. A 12-mm assistant port is placed in the mid-line in either the upper or lower quadrant, depending on tumor location and surgeon preference. If a fourth robotic arm is used, an additional 8-mm trocar is placed laterally, triangulated between the two other robotic trocars (Cabello et al., 2009; Kavoussi et al., 2011). The robot is docked posterior to the patient.

Access for retroperitoneal RAPN is obtained through a 1.2-cm skin incision just inferior to the tip of the 12th rib. The flank muscle fibers and thoracolumbar fascia are bluntly split, and the surgeon’s fingertip creates a potential space between the psoas muscle and Gerota’s fascia; this space is further expanded by injection of 800 mL of air into the retroperitoneum through a balloon dilator. The camera port is then placed at the site of the balloon dilator. Generally, only two additional working ports, triangulated with the camera at an obtuse angle to reduce instrument collisions, are required. If a fourth robotic arm is used, the peritoneum is pushed medial to the belly of the rectus in order to expand the retroperitoneal workspace, and the fourth arm is placed anteriorly in that location. An assistant port is placed in the lower quadrant (Rogers, 2009; Dulabon and Stifelman, 2011). The robot is docked anterior to the patient.

## SURGERY

Depending on tumor location, patient surgical history, and surgeon preference, a transperitoneal or retroperitoneal approach is selected. While safe and effective in experienced hands, the retroperitoneal approach is potentially more challenging due to its confined workspace and relatively fewer anatomic landmarks (Weizer et al., 2011).

### LESION EXCISION

Following tumor exposure, its precise borders are delineated, often under intraoperative ultrasound guidance. The newly developed robot-controlled ultrasound probe (Aloka, Tokyo, Japan) allows full surgeon control of intraoperative imaging. TilePro software integration, included in newer robotic platforms, allows for real-time picture-on-picture display of radiographic images on the console screen, thus facilitating the mapping out of the dissection.

Traditionally, renal hilar vessels are clamped – either individually (starting with the artery) using laparoscopic bulldog clamps or *en bloc* using a laparoscopic Satinsky clamp – prior to tumor excision; the latter requires placement of a dedicated port. Recently developed “robotic bulldog clamps” provide the surgeon additional autonomy, in lieu of having to relegate the delicate task of hilar occlusion to the assistant. The tumor is excised sharply with a rim of normal renal parenchyma. The assistant applies counter-traction with the suction device to enhance visualization during tumor excision.

### REPAIR

Using robotic needle drivers, renorrhaphy is traditionally performed in two layers. A deep-layer closure of the resection bed, which includes repair of large blood vessels and collecting system defects, is first performed with a poliglecaprone 25 or polyglactin suture in a running fashion. This is followed by an outer-layer closure of the renal capsule, performed using larger absorbable sutures and needles. The Washington University technique of “sliding-clip renorrhaphy” – widely adopted as a preferable alternative to the traditional tied-suture renorrhaphy – relies upon the use of Weck Hem-o-Lok clips, placed on either side of the defect and then slid into place by the surgeon, to exert tension upon the repair. The Hem-o-Lok clips are generally reinforced with LapraTy clips to prevent backsliding of the clips. This technique is ideally suited for RAPN, as the robotic instrumentation affords the surgeon the requisite precision in dictating the degree of tension placed on the repair, effectively eliminating the need for placement of surgical bolsters in the renal defect to achieve tight closure (Benway et al., 2009c).

### TECHNIQUES TO MINIMIZE WARM ISCHEMIA

As minimally invasive techniques for achieving renal hypothermia during renal hilar clamping have failed to gain widespread clinical application, RAPN is generally performed under conditions of “warm ischemia.” In recognition of the potential adverse effect that even limited warm ischemia time (WIT) may have on kidney function (Lane et al., 2008; Becker et al., 2009; Thompson et al., 2010), multiple investigators have compared WIT during RAPN vs. LPN. While several earlier studies found no significant difference in WIT between RAPN and LPN (Caruso et al., 2006; Aron et al., 2008; Deane et al., 2008), a recent study comparing 129 patients who underwent RAPN and 118 patients who underwent LPN demonstrated significantly reduced WIT in the RAPN group (19.7 vs. 28.4 min, *p* < 0.001); RAPN maintained consistently shorter WIT than LPN in patients with “complex” renal tumors (25.9 vs. 36.7 min, *p* < 0.001; Benway et al., 2009b). Other smaller comparative series of RAPN and LPN have also demonstrated a reduction in WIT with RAPN (Wang and Bhayani, 2008; Williams et al., 2011). Importantly, individual surgeon experience significantly impacts WIT independent of surgical technique. Nonetheless, preliminary investigation suggests that RAPN may not require as long a learning curve as LPN to achieve a reasonable WIT (Mottrie et al., 2010). WIT in multiple RAPN series are presented in **Table 1** (Gettman et al., 2004; Bhayani and Das, 2008; Rogers et al., 2008; Benway et al., 2010; Mottrie et al., 2010; Patel et al., 2010; Scoll et al., 2010; Dulabon et al., 2011; Lorenzo et al., 2011; Naeem et al., 2011; Petros et al., 2011; Simhan et al., 2012), and studies comparing WIT during RAPN vs. LPN are outlined in **Table 2** (Caruso et al., 2006; Aron et al., 2008; Deane et al., 2008; Wang and Bhayani, 2008; Benway et al., 2009b; Haber et al., 2010; Williams et al., 2011).

**Table 1:** Contemporary robotic partial nephrectomy case series.

Reference	No. of cases	Mean tumor size (cm)	Mean OR time (min)	Mean, WIT (min)	Mean EBL (mL)	PSM, *n* (%)	Complications, *n* (%)	Mean LOS (day)	Mean, f/u (month)
Gettman et al. (2004)	13	NR	215	22	170	1 (7.7)	NR	4.3	NR
Bhayani and Das (2008)	35	2.8	142	21.0	133	None	6 (17)	2.5	NR
Rogers et al. (2008)	148	2.8	197	27.7	183	6 (4)	9 (6)	1.9	18
Mottrie et al. (2010)	62	2.8	90-median	20	95-median	2 (3.2)	10 (16.1)	5-median	NR
Benway et al. (2010)	183	2.9	210	23.9	132	7 (3.8)	18 (9.8)	NR	≤26
Scoll et al. (2010)	100	2.8	206	25.5	127	5 (5.7)	13 (13)	3.2	12.7
Patel et al. (2010)	71	2.1–5	238–275	20–25	100	3 (4.2)	9 (12.6)	2	12
Petros et al. (2011)	95	2.3–2.5	246–250	16–21	100–150	NR	22 (23)	2	NR
Lorenzo et al. (2011)	65	NR	171	NR	243.2	6 (9.2)	1 (1.5)	4.6	13
Naeem et al. (2011)	97	2.3–2.5	243–265	22.5–26.5	100–150	2 (2)	8 (8.2)	2	12
Dulabon et al. (2011) (non-hilar vs. hilar)	405 vs. 41	2.9 vs. 3.5	187.4 vs. 194.5	19.6 vs. 26.3	208.2 vs. 262.2	6 (1.5) vs. 1 (2.4)	22 (5.4) vs. 1 (2.4)	2.9 vs. 2.9	≤45
Simhan et al. (2012) (“moderately complex” tumors)	81	3.2	205.9	NR	131.3	3 (3.7)	NR	3.7	NR

*WIT, warm ischemia time, EBL; estimated blood loss; LOS, length of stay; PSM, positive surgical margin; f/u, follow-up; NR, not reported.*

**Table 2:** Series comparing RAPN vs. LPN.

Reference	No. of cases	Mean tumor size (cm)	Mean OR time (min)	Mean WIT (min)	Mean EBL (mL)	PSM, *n* (%)	Complications, *n* (%)	Mean LOS (day)	Mean f/u (month)
Caruso et al. (2006)	RAPN: 10 LPN: 10	RAPN: 2.0 LPN: 2.2	RAPN: 279 LPN: 253	RAPN: 26.4 LPN: 29.3	RAPN: 240 LPN: 200	RAPN: 0 (0) LPN: 1 (10)	RAPN: 1 (10) LPN: 1 (10)	RAPN: 2.6 LPN: 2.7	RAPN: NR LPN: NR
Aron et al. (2008)	RAPN: 12 LPN: 12	RAPN: 2.4 LPN: 2.9	RAPN: 242 LPN: 256	RAPN: 23 LPN: 22	RAPN: 329 LPN: 300	RAPN: NR LPN: NR	RAPN: NR LPN: NR	RAPN: 4.7 LPN: 4.4	RAPN: 7.4 LPN: 8.5
Deane et al. (2008)	RAPN: 11 LPN: 11	RAPN: 3.1 LPN: 2.3	RAPN: 229 LPN: 290	RAPN: 32.1 LPN: 35.3	RAPN: 115 LPN: 198	RAPN: 0 (0) LPN: 1 (9.1)	RAPN: 1 (9.1) LPN: 1 (9.1)	RAPN: 2.0 LPN: 3.1	RAPN: 16 LPN: 4.5
Benway et al. (2009b)	RAPN: 129 LPN: 118	RAPN: 2.9 LPN: 2.6	RAPN: 189 LPN: 174	RAPN: 19.7 LPN: 28.4	RAPN: 155 LPN: 196	RAPN: 5 (3.9) LPN: 1 (0.8)	RAPN: 11 (8.5) LPN: 12 (10.2)	RAPN: 2.4 LPN: 2.7	RAPN: NR LPN: NR
Wang and Bhayani (2008)	RAPN: 40 LPN: 62	RAPN: 2.5 LPN: 2.4	RAPN: 140 LPN: 156	RAPN: 19 LPN: 25	RAPN: 137 LPN: 173	RAPN: 1 (2.5) LPN: 1 (1.6)	RAPN: 8 (20) LPN: 9 (14.5)	RAPN: 2.5 LPN: 2.9	RAPN: NR LPN: NR
Haber et al. (2010)	RAPN: 75 LPN: 75	RAPN: 2.8 LPN: 2.5	RAPN: 200 LPN: 197	RAPN: 18.2 LPN: 20.3	RAPN: 323 LPN: 222	RAPN: 0 (0) LPN: 0 (0)	RAPN: 12 (16.0) LPN: 10 (13.3)	RAPN: 4.2 LPN: 4.1	RAPN: NR LPN: NR
Williams et al. (2011)	RAPN: 27 LPN: 59	RAPN: 2.5 LPN: 3.1	RAPN: 233 LPN: 221	RAPN: 18.5 LPN: 28.0	RAPN: 180 LPN: 146	RAPN: 1 (3.7) LPN: 7 (11.9)	RAPN: 5 (18.5) LPN: 12 (20.3)	RAPN: 2.5 LPN: 2.7	RAPN: NR LPN: NR

*RAPN, robot-assisted partial nephrectomy; LPN, laparoscopic partial nephrectomy; WIT, warm ischemia time; EBL, estimated blood loss; LOS, length of stay; PSM, positive surgical margin; f/u, follow-up; NR, not reported.*

By simplifying the renorrhaphy, while minimizing reliance on the surgical assistant, the sliding-clip technique, as described above, has been shown to substantially reduce WIT during RAPN, compared to tied-suture renorrhaphy (Benway et al., 2009c). Precluding the need for additional “anchoring” LapraTy clips, the more recent application of barbed suture to facilitate tight parenchymal closure during sliding-clip renorrhaphy may further decrease WIT (Sammon et al., 2011; Sukumar and Rogers, 2011).

Other variations in surgical technique previously described for OPN and LPN have also been applied to RAPN in an effort to minimize or eliminate WIT. Some have adopted an “early unclamping technique,” clamping the renal hilum only during tumor resection and while suturing large vessels/collecting system at the resection base (San Francisco et al., 2011). Others have performed “selective renal parenchymal clamping,” clamping regional blood vessels only in the area of planned excision (Figenshau, 2005; Benway et al., 2009a). More recently, some have performed RAPN without any clamping of the renal hilum, suggesting that off-clamp RAPN can be safely performed in carefully selected patients (Wu et al., 2010; Tanagho et al., 2012a,b). Our initial experience with off-clamp RAPN at Washington University demonstrates favorable renal functional outcomes with this technique (Tanagho et al., 2012a,b). Nevertheless, several studies have failed to demonstrate long-term improvement in renal functional outcomes after any modification of clamping technique or in the absence of renal hilar clamping (Bhayani et al., 2004; Foyil et al., 2008; Becker et al., 2009). Further studies will be needed to establish the efficacy and reaffirm the safety of these surgical approaches.

## COMPLICATIONS

Early series of RAPN reported complication rates ranging from 0 to 20% (Cha et al., 2011). A contemporary study of 886 consecutive cases of RAPN performed at five U.S. centers reported an overall complication rate of 15.6%, with intraoperative and postoperative complication rates of 2.6 and 13.0%, respectively. Postoperative complications were classified as Clavien grade I–II in 77.0% of cases and grade III–IV in 23.0%. RAPN was converted to OPN or LPN in 0.2% of patients and to RN in 0.5% of patients. There were no deaths (Tanagho et al., 2012c). **Table 1** summarizes complication rates of various RAPN series (Gettman et al., 2004; Bhayani and Das, 2008; Rogers et al., 2008; Benway et al., 2010; Mottrie et al., 2010; Patel et al., 2010; Scoll et al., 2010; Dulabon et al., 2011; Lorenzo et al., 2011; Naeem et al., 2011; Petros et al., 2011; Simhan et al., 2012).

Reported complication rates of RAPN are comparable to those seen in OPN and LPN. For example, Gill et al. (2007) reported complications in 13.7 and 18.6% of patients undergoing OPN and LPN, respectively. A study by Simhan et al. (2012) comparing outcomes of RAPN vs. OPN in 281 patients demonstrated similar major and minor complication rates in the two groups. Benway et al. (2009b) compared 129 patients who underwent RAPN with 118 patients who underwent LPN and found no significant differences in complication rates. Series comparing complication rates of RAPN and LPN are presented in **Table 2** (Caruso et al., 2006; Aron et al., 2008; Deane et al., 2008; Wang and Bhayani, 2008; Benway et al., 2009b; Haber et al., 2010; Williams et al., 2011).

In the following section, some of the more common and significant complications of RAPN are discussed.

### HEMORRHAGE

#### Intraoperative

Tanagho et al.’s (2012c) multi-institutional analysis of RAPN complications demonstrated an intraoperative hemorrhage rate of 1.0%. (Hemorrhage was defined as bleeding requiring blood transfusion or therapeutic intervention.) Parenchymal bleeding during tumor excision may result from inadequate hilar occlusion or unrecognized accessory vessels. Pulsatile bleeding of a typically larger volume indicates an arterial source, while a lower volume ooze suggests a venous source. In the case of arterial bleeding, controlled anesthetic blood pressure reduction may enhance visualization, and temporary removal of the venous clamp may reduce parenchymal congestion. *En bloc* hilar clamping with a Satinsky clamp or a long bulldog clamp is another option. Venous bleeding can often be mitigated by raising the pneumoperitoneum up to 18 mmHg. In contrast to the renal vein unclamping maneuver recommended for arterial bleeding, with venous bleeding, an additional clamp may be placed on the renal vein to minimize venous backflow. Inadequate sutured renal reconstruction may lead to bleeding after the renal hilum is unclamped. Direct pressure is immediately applied and the insufflation pressure increased while the renorrhaphy clips are re-tightened. Emergent open conversion or conversion to robotic total nephrectomy may be necessary for uncontrolled bleeding (Nepple et al., 2012).

#### Postoperative

In our multi-institutional analysis of RAPN complications, we reported a postoperative hemorrhage rate of 5.8% (Tanagho et al., 2012c). Published postoperative transfusion rates for RAPN range from 3 to 10%, which are comparable to the 5.8 and 3.4% rates for LPN and OPN, respectively (Cha et al., 2011). Pseudoaneurysm or arteriovenous fistula formation may result in delayed postoperative hemorrhage, often presenting several weeks after discharge (Benway et al., 2009b). Angiography and embolization are indicated for persistent bleeding and/or hemodynamic instability.

### URINE LEAK

Although initial urinary leak rates reported for RAPN ranged from 2 to 12.5% (Cha et al., 2011), Tanagho et al.’s (2012c) more contemporary multi-center study of RAPN complications reported urine leakage (defined as urine extravasation identified radiographically or “persistently” increased drain fluid creatinine) in only 1.1% of cases; this rate is significantly lower than the 3.1 and 2.3% rates reported in LPN series (American Urological Association Education and Research, 2009; Cha et al., 2011). In the absence of obstruction distal to the leakage site, the majority of urine leaks will spontaneously resolve within several weeks. Retrograde placement of a ureteric stent may be indicated if conservative management fails (Meeks et al., 2008).

## RESULTS

Taken together, single series (**Table 1**; Gettman et al., 2004; Bhayani and Das, 2008; Rogers et al., 2008; Benway et al., 2010; Mottrie et al., 2010; Patel et al., 2010; Scoll et al., 2010; Dulabon et al., 2011; Lorenzo et al., 2011; Naeem et al., 2011; Petros et al., 2011; Simhan et al., 2012) and comparative studies (**Table 2**; Caruso et al., 2006; Aron et al., 2008; Deane et al., 2008; Wang and Bhayani, 2008; Benway et al., 2009b; Haber et al., 2010; Williams et al., 2011) have demonstrated that RAPN can be performed safely and with acceptable oncological, functional, and perioperative outcomes. Nonetheless, carefully matched (ideally, randomized) comparisons of OPN, LPN, and RAPN with long-term follow-up are still required. The recent development of metrics for comparing renal mass complexity (e.g., R.E.N.A.L. nephrometry score, Kutikov and Uzzo, 2009; and PADUA score, Ficarra et al., 2009) may facilitate such comparisons.

### ONCOLOGIC OUTCOMES

Because RAPN is a novel and maturing technique, positive surgical margin (PSM) rates have often been reported as a surrogate for oncological control. A review of contemporary RAPN series demonstrated a cumulative PSM rate of 2.7% (Benway and Bhayani, 2011), which is comparable to the 2.9 and 1.3% rates previously reported for LPN and OPN, respectively (Gill et al., 2007). PSM rates in various RAPN series are depicted in **Table 1** (Gettman et al., 2004; Bhayani and Das, 2008; Rogers et al., 2008; Benway et al., 2010; Mottrie et al., 2010; Patel et al., 2010; Scoll et al., 2010; Dulabon et al., 2011; Lorenzo et al., 2011; Naeem et al., 2011; Petros et al., 2011; Simhan et al., 2012), while studies comparing PSM rates between RAPN and LPN are outlined in **Table 2** (Caruso et al., 2006; Aron et al., 2008; Deane et al., 2008; Wang and Bhayani, 2008; Benway et al., 2009b; Haber et al., 2010; Williams et al., 2011).

Early and intermediate outcomes of RAPN show excellent oncological control (Kyllo et al., 2012). In fact, a review of modern large RAPN series encompassing >1600 patients demonstrated only seven recurrences, a rate of <1% (Benway and Bhayani, 2011). Although these early reports are certainly encouraging, long-term data on RAPN are presently lacking. Long-term oncological outcomes from the largest series of LPN were recently reported and were comparable with those of OPN (Lane and Gill, 2010). Several recent studies have shown that the oncological outcomes of RAPN are equivalent to those of LPN in the short and intermediate term (Aron et al., 2008; Rogers et al., 2008; Wang and Bhayani, 2008; Patel et al., 2010; Dulabon et al., 2011).

### RENAL FUNCTIONAL OUTCOMES

Overtreatment with RN has been linked to increased risk of chronic renal insufficiency and higher mortality (Huang et al., 2006; Thompson et al., 2008; Weight et al., 2010). A contemporary series of patients with unanticipated benign tumors demonstrated a 2.5-fold increased mortality 5 years following RN, compared to PN (open or laparoscopic; Weight et al., 2010). The clear advantage of NSS in preserving renal function which numerous OPN and LPN series have shown has been similarly demonstrated in series of RAPN. Indeed, an international, multi-center study of 183 patients showed no significant postoperative change in estimated glomerular filtration rate (eGFR; 82.2 vs. 79.4 mL/min/1.73 m^2^, *p* = 0.74) up to 26 months following RAPN (Benway et al., 2010). Simhan’s study comparing outcomes of RAPN vs. OPN demonstrated similar percent changes in eGFR in the two groups (Simhan et al., 2012). Studies comparing renal functional outcomes of RAPN vs. RN and RAPN vs. LPN are still needed.

### PERIOPERATIVE OUTCOMES

Early studies by Caruso, Deane, and Aron reported no significant differences in operative time or EBL between RAPN and LPN (Caruso et al., 2006; Aron et al., 2008; Deane et al., 2008). Similarly, in a matched cohort study of 150 patients undergoing RAPN or LPN, Haber et al. (2010) found no significant difference in operative time and LOS.

To the contrary, Benway et al. (2009b) reported a significant decrease in EBL (155 vs. 196 mL, *p* = 0.03) and LOS (2.4 vs. 2.7 days, *p* < 0.001) in patients undergoing RAPN vs. LPN, respectively. Wang and Bhayani (2008) also reported a shorter operative time (140 vs. 156 min, *p* = 0.04) and LOS (2.5 vs. 2.9 days, *p* = 0.03) for RAPN in their series of 40 RAPNs and 62 LPNs. **Table 1** depicts perioperative outcomes of various RAPN series (Gettman et al., 2004; Bhayani and Das, 2008; Rogers et al., 2008; Benway et al., 2010; Mottrie et al., 2010; Patel et al., 2010; Scoll et al., 2010; Dulabon et al., 2011; Lorenzo et al., 2011; Naeem et al., 2011; Petros et al., 2011; Simhan et al., 2012), while studies comparing perioperative outcomes of RAPN vs. LPN are summarized in **Table 2** (Caruso et al., 2006; Aron et al., 2008; Deane et al., 2008; Wang and Bhayani, 2008; Benway et al., 2009b; Haber et al., 2010; Williams et al., 2011).

## CONCLUSION

Despite the growing acknowledgment of elective PN as a feasible, oncologically sound, and less morbid treatment option for managing SRM, PN remains grossly underutilized, particularly in the community setting (Cooperberg et al., 2011). In the absence of adequate ancillary health services, the greater technical complexity and higher risk of vascular and urinary complications associated with PN (American Urological Association Education and Research, 2009) may dissuade general urologists from performing this procedure electively. Furthermore, given the potential advantages and popularization of minimally invasive surgery over open surgery, the advanced level of technical proficiency required to perform LPN may deter some surgeons from performing a minimally invasive nephron-sparing procedure in favor of the less technically demanding laparoscopic RN.

Surmounting some of the technical challenges associated with LPN (Deane et al., 2008; Mottrie et al., 2010), the emergence of RAPN as a minimally invasive nephron-sparing alternative to LPN may play a critical role in facilitating the wider dissemination of NSS into general practices. Indeed, with growing expertise in robotic surgery, RAPN has been offered to an increasing number of patients, including those with larger, endophytic, and central masses (Cha et al., 2011); moreover, studies have demonstrated that RAPN can, in fact, be performed safely and with acceptable outcomes for renal tumors of increasingly greater complexity (Rogers et al., 2008; Patel et al., 2010; Dulabon et al., 2011). Comparative studies of RAPN vs. LPN have also demonstrated favorable outcomes for RAPN (Caruso et al., 2006; Aron et al., 2008; Deane et al., 2008; Wang and Bhayani, 2008; Benway et al., 2009b; Haber et al., 2010; Williams et al., 2011). Nonetheless, long-term data on RAPN – essential for the full espousal of this technique – are presently lacking.

As a nascent procedure, the technique of RAPN continues to evolve. Further advances in minimizing WIT and facilitating the use of cold ischemia are expected. Technical innovation in robotic instrumentation may also enhance the technique of RAPN. For example, the development of systems such as TilePro, which enable picture-on-picture display of radiographic images on the console screen, may facilitate precise tumor dissection. The addition of the fourth robotic arm in the da Vinci S and Si systems decreases the surgeon’s dependence on the bedside assistant during retraction, dissection, and reconstruction. Furthermore, with the introduction of the robotic ultrasound probe, robotic bulldog clamps, and barbed suture, the trend for maximizing the autonomy of the console surgeon during the critical steps of tumor identification, hilar clamping, and renorrhaphy is becoming increasingly apparent. The future design of robotic systems capable of providing tactile feedback to the surgeon may also contribute to the safety and efficacy of RAPN, particularly for the management of complex renal masses.

## Conflict of Interest Statement

Sam B. Bhayani is a consultant for Surgi Quest, Inc. Youssef S. Tanagho and Robert S. Figenshau have no conflicts of interest.

## Abbreviations

EBL: estimated blood loss
eGFR: estimated glomerular filtration rate
LOS: length of hospital stay
LPN: laparoscopic partial nephrectomy
NSS: nephron-sparing surgery
OPN: open partial nephrectomy
PN: partial nephrectomy
PSM: positive surgical margin
RAPN: robot-assisted partial nephrectomy
RN: radical nephrectomy
SRM: small renal masses
WIT: warm ischemia time.
